# T2-Imaging of the ischemic area-at-risk predicts recovery of cardiac function after acute ST-elevation myocardial infarction

**DOI:** 10.1186/1532-429X-14-S1-P26

**Published:** 2012-02-01

**Authors:** Jamieson M Bourque, Emily A Pearce, Ashul Govil, Anshul Aggarwal, Amit Bhaskar, Christopher M Kramer

**Affiliations:** 1 Radiology, University of Virginia Health System, Charlottesville, VA, USA; 2 Medicine, University of Virginia Health System, Charlottesville, VA, USA

## Background

T2-weighted edema imaging identifies the ischemic area at risk during acute myocardial infarction. Myocardial salvage occurs in the area at risk without late gadolinium enhancement (LGE). The degree of myocardial salvage is prognostically important, but its effect on recovery of left ventricular (LV) function is not known. The purpose of this study was to determine if the degree of myocardial salvage predicts recovery of function after an acute ST-elevation myocardial infarction (STEMI).

## Methods

We assessed patients with no known prior CAD post-STEMI. Imaging was performed within 72 hours of the acute event and was then repeated 6-12 weeks post-infarction. The degree of myocardial salvage was obtained by subtracting the percentage LGE from the T2 area at risk. The degree of salvage was compared to the percentage improvement in LVEF by nonparametric Spearman rank correlation coefficient analysis.

## Results

Twenty-three patients were recruited for the study. One patient refused a second study, 2 had technical difficulties, and 5 had a baseline LV ejection fraction (EF) of ≥50%, leaving a final study population of 15 subjects. The mean age of the sample was 59.3 ± 11.7 years; 86.7% were male and 93.3% were Caucasian. Hypertension, hyperlipidemia, and tobacco use was present in 40.0%, 73.3%, and 46.7%, respectively. The mean BMI was 30.8 ± 4.1, and 9/15 (60%) had a BMI ≥30. The mean LDL was 118.7 ± 33.0, and the mean HgA1c was 6.7 ± 1.8. The baseline mean LVEF was 42.1 ± 6.6%, and increased to 45.1 ± 9.8%, a mean increase of 3.0%. The mean percentage T2 enhancement was 41.2 ± 17.4%, and the mean burden of LGE was 23.5 ± 14.0%, giving a percentage myocardial salvage of 17.7%. The degree of myocardial salvage correlated moderately with an improvement of LVEF from the acute to recovery CMR, with a Spearman correlation coefficient of 0.59 (p= 0.020).

## Conclusions

The percentage of myocardial salvage moderately predicts recovery of function after an acute STEMI. Future analysis will focus on identifying additional predictors on post-infarction cardiac magnetic resonance imaging.

## Funding

None.

